# Assessment of an original dental autotransplantation technique: A retrospective study and proposal of a method

**DOI:** 10.4317/jced.62038

**Published:** 2024-10-01

**Authors:** Amira Hadji, Gérard Bader

**Affiliations:** 1 Resident in oral surgery, Department of Oral Surgery, Hospital Center of Rennes, Pontchaillou Hospital, 35000 Rennes, France; 2 Oral surgeon, Department of Oral Surgery, Hospital Center of Rennes, Pontchaillou Hospital, 35000 Rennes, France

## Abstract

**Background:**

Successful tooth autotransplantation (TAT) requires preservation of the periodontal ligament (PDL) on donor teeth and good vascularization of the recipient site to ensure maintenance of alveolar bone volume through physiological stimulation of PDL. This retrospective study aims to evaluate the survival and success rate of a two-step procedure that uses dual periodontal stimulation and a 3D replica of the transplanted tooth to promote ligament repair and prevent ankylosis and root resorption.

**Material and Methods:**

All consecutive patients followed at the dental center of Rennes and having undergone a TAT in two surgical stages with double periodontal stimulation and the use of a 3D replica of the transplanted tooth between 2017 and 2022 were invited for follow-up clinical and radiographic examination. First, survival rates were calculated on the basis of a telephone survey. Then, a clinical and radiological follow-up examination was used to calculate the success rate.

**Results:**

Of these 22 transplants, 21 were still functioning, and 1 had been extracted, giving a 95,5% probability of survival after a median follow-up of 23 month. Of the 21 teeth eligible for success analysis, clinical and radiological follow-up showed a success rate of 90,5% with normal PDL and no ankylosis.

**Conclusions:**

The teeth auto-transplanted by this procedure gave a very satisfactory survival and success rate in the medium term. This study suggests that this standardized autotransplantation procedure potentiates PDL healing and may be a viable and predictable treatment in current clinical practice, especially when orthodontic treatment is required.

** Key words:**Tooth autotransplantation, 3D printing, periodontal regeneration, bone regeneration.

## Introduction

Autotransplantation offers a significant advantage in forming a periodontal ligament (PDL) around the transplanted tooth, particularly crucial in children and adolescents for preserving and promoting continuous growth of the alveolar crest. Scientific advancements in pulp and periodontal healing over the past three decades have led to modern autotransplantation techniques, aiming for improved outcomes (1). Nethander *et al*. proposed transplanting teeth into alveoli with regenerative tissues to enhance nutrition and preserve PDL cellular activity, reducing complications like root resorption and ankylosis (2-4). Improved nourishment is achieved by transplanting the donor tooth into the vascularized connective tissue of a healing alveolus after a 14-day interval (5).

Gault *et al*. demonstrated that mechanical stimulation of PDL cells before autotransplantation induces a reactive proliferation of fibroblasts on the root surface, optimizing PDL healing (6). To prevent damage to the alveolo-dental ligament during tooth transplantation, our protocol incorporates cone beam computed tomography (CBCT) and computer-aided manufacturing design (CAD/CAM) to produce 3D dental replicas. These replicas serve as guides, minimizing extra-oral time during surgery and reducing harm to PDL cells (7-9).

This study introduces an innovative, standardized dental autotransplantation technique involving double periodontal stimulation and the use of 3D dental replicas. The objective is to evaluate the success and survival rate of teeth autotransplanted using this two-stage surgical approach.

## Material and Methods

-Study design

In this retrospective, single-center study, all consecutive patients who underwent a two-stage autotransplantation procedure with dual periodontal stimulation and use of a 3D replica of donor tooth at the Rennes Dental University Hospital (RDUH) between March 2017 and September 2022 were identified through a computerized search of patient records at the RDUH and included in the study. Patients who underwent a one-stage autotransplantation procedure and did not use a 3D replica of the donor tooth were excluded from the study. Subsequently, they were contacted by phone. All contacted patients received detailed information about the current study and were invited for follow-up examination. The 22 transplanted teeth were retrospectively examined. None of the participants had any systemic diseases that could have led to infections and/or affected the healing of soft tissues or bone formation. The study was conducted in accordance with the institutional and national ethical standards of the responsible committee on human experimentation and with the principles of the Declaration of Helsinki.

-Operative procedure

1. Manufacture of the 3D replica

CBCT imaging and CAD/CAM offer innovative possibilities for autotransplantation (10). This 3D additive manufacturing technology involves building a three-dimensional object directly from a 3D model in any file format (e.g., STL, STP, 3MF) (11). It enables individualized preparation of the new dental socket (neo-alveolus) prior to donor tooth extraction (12). This technique minimizes the risk of iatrogenic damage to donor teeth during multiple attempts to adjust the recipient site, reducing extra-alveolar time and manipulation of the PDL of the donor teeth, which is a key factor for successful autotransplantation. Additionally, using a replica of the transplanted tooth helps standardize the autotransplantation procedure (13).

**Figure 1:**
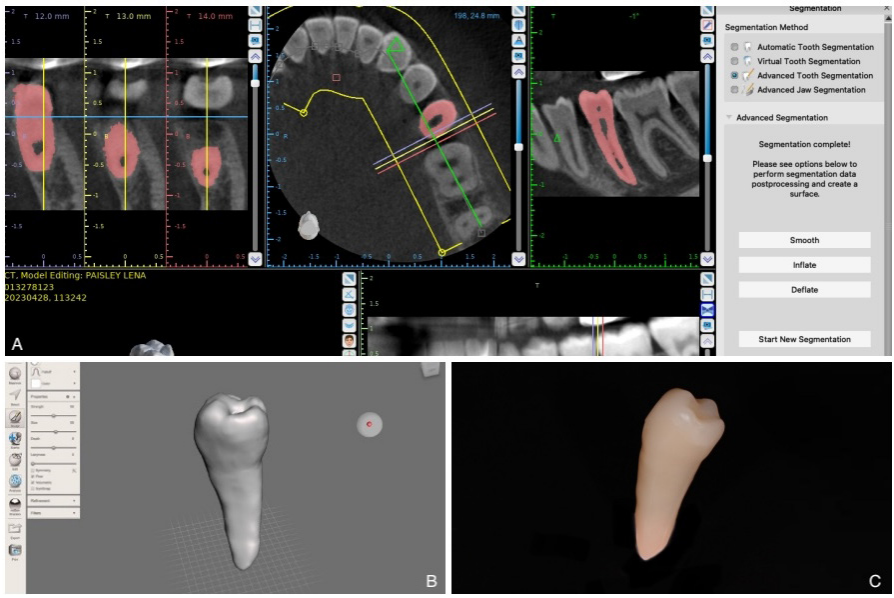
Digital planning and 3D-printed CARP models of the donor tooth. A) Extraction of the dental volume from CBCT in STL format and segmentation of the donor tooth #35 (in red) on Blue Sky Plan®. B) Thesmoothing of the volume obtained by additions on Meshmixer. C) Machined dental replica of tooth 35.

To obtain the replica, CBCT images were used. After obtaining the DICOM-formatted image, it was segmented using Blue Sky Plan® software. This software allows the separation of a digital image into different structures by selecting the anatomical elements concerned (Fig. 1a). Once the images were digitally segmented, in STL format, the removal of artifacts around the STL image of the donor tooth was performed using Meshmixer software (Autodesk®), a free 3D general design software (Fig. 1b). Then, the STL image of the donor tooth was sent to a 3D printer for its manufacture with biocompatible resin (Fig. 1c). Before surgery, the surgical replicas of the donor tooth were sterilized.

2. Two-stage surgical technique

**Figure 2:**
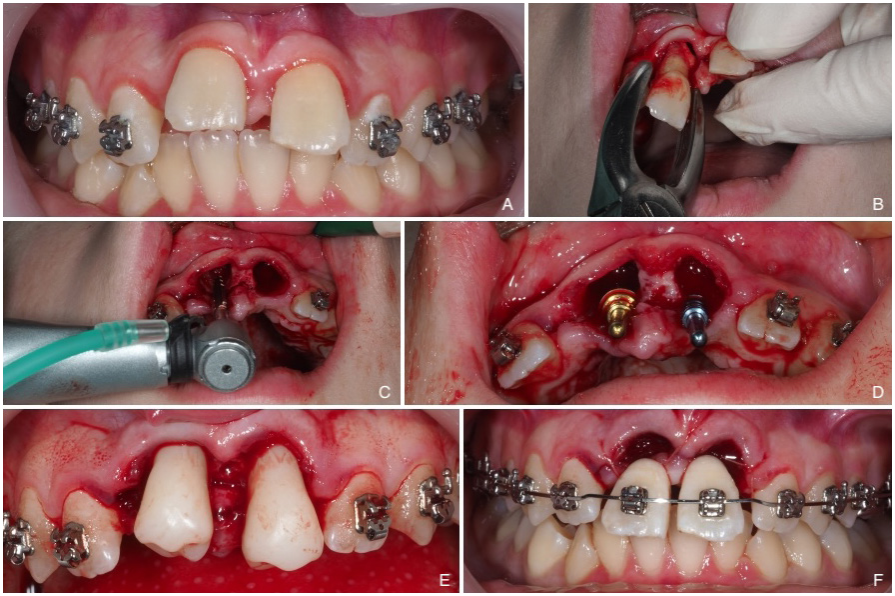
First stage of auto-transplantation of #35 and 45 to #11 and 21 recipient sites. A) #11 and 21 in infraclusion following trauma. B) Extraction of teeth #11 and 21. C,D) Preparation of recipient sites using implant drills and implant parallelizers. E) Fitting of dental replicas. F) Use of crowns #11 and 21 extracted and arched to ensure aesthetics.

First Surgical Step:

Under local anesthesia (LA), teeth at the recipient site are atraumatically removed to preserve the vestibular cortical (Fig. 2a). Using a 3D replica of the donor tooth and an implant drilling kit, the recipient site is prepared, ensuring minimal pressure on the tissue for microcirculation and bone regeneration (Fig. 2b-d) The flap is repositioned and sutured (Fig. 2e,f). If recovery is incident-free, the second surgical stage is performed after 14 days.

**Figure 3:**
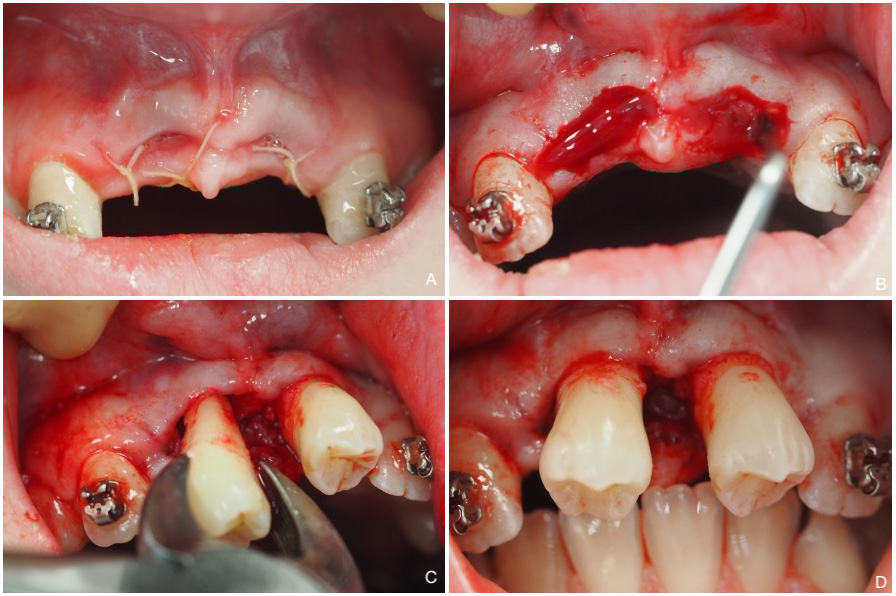
Second stage of auto-transplantation of #35 and 45 to #11 and 21 recipient sites. A) Recipient sites at 14 days. B) Incision and elimination of coagulum. C) Extraction and immediate transplantation of #35 and #45. D) Occlusion check.

Second Surgical Stage:

Tissues around the recipient and donor sites are infiltrated with LA (Fig. 3a). At the recipient site, wound margins and the upper part of the coagulum are excised to remove the entire epithelium (Fig. 3b). The donor tooth is atraumatically extracted and immediately transplanted, ensuring no contact with the root surface during the procedure (Fig. 3c). Gingiva is sutured closely to the transplant with absorbable thread, and occlusion is controlled to verify the absence of overbite (Fig. 3d).

3. Double periodontal ligament stimulation

During the initial surgical phase under local anesthesia, double stimulation of the periodontal ligament is achieved by delicately mobilizing the donor tooth in its site using rotational movements. Controlled tissue trauma is applied to induce a reactive proliferation of periodontal ligament fibroblasts on the root surface, and the tooth is transplanted along with these cells after a 14-day interval. This biological stimulation aids in managing the inflammatory process and accelerates the healing kinetics of the periodontal ligament, minimizing the impact of the initial inflammatory destruction phase while promoting ligament healing around the transplanted root through the presence of a high density of activated fibroblasts on its surface (6).

**Figure 4:**
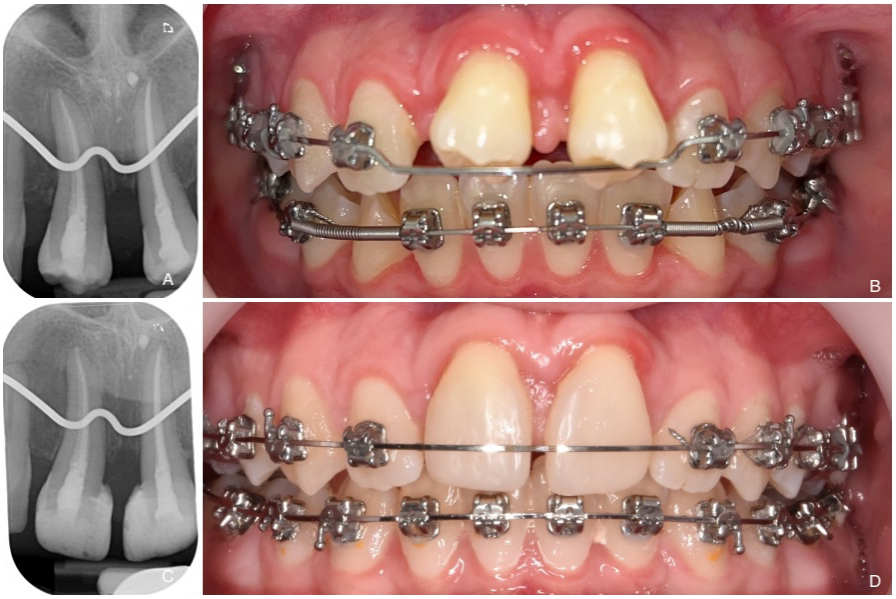
Post-transplant follow-up. A,B) Radiographic X-ray and photography of transplants #35 and #45 to #11 and #21 recipient site one month after TAT. C,D) Radiographic X-ray and photography of transplants #35 and #45 to #11 and #21 recipient site fourteen month after TAT.

Subsequently, a second periodontal ligament stimulation is employed to regulate the competition between periodontal ligament (PDL) and bone tissues, favoring fibroblast cells over osteoblast cells. This is achieved post-transplantation through a flexible immobilization technique using sutures to prevent the formation of bone bridges, which can lead to ankylosis. The split of the transplanted tooth is removed two weeks after autotransplantation. Follow-up assessments are conducted at 1 month (Fig. 4a,b), 3 months, 6 months, and then annually (Fig. 4c,d).

-Data acquisition and analysis

1. Phone call 

All patients were asked to provide information about the status of the transplant upon phone inquiry, i.e., if the tooth was still in place, if any additional treatment had been performed so far, if any symptom was present in rest or function, as well as subjective satisfaction with TAT treatment.

2. Clinical and radiographic evaluation

Patients who agreed to attend a follow-up examination were evaluated clinically and radiographically by the principal investigator (AH).

Clinical assessments for transplanted tooth healing included tooth mobility (Mühlemann index), percussion tests, pulp vitality, sulcular bleeding index, probing pocket depth, recession, attachment loss, percussion pain, signs of apical periodontitis, occlusion type, and the presence of an artificial crown. Masticatory function was considered normal if patients could chew without pain. Radiological evaluation utilized periapical radiographs to assess root formation stages and healing criteria for PDL and pulp. The presence of radiolucent periapical images, intra- and peri-radicular images, and lamina dura indicated PDL healing. Ankylosis or root resorption diagnosis was made within three months post-intervention (14). Pulp healing was determined by pulp cavity obliteration or root length progression. Lack of response to pulp sensitivity tests was disregarded if radiographic evidence of healing was observed within the first six months post-transplantation. Vertical bone healing was assessed by comparing preoperative and postoperative bone levels around the transplanted tooth after six months.

-Survival and clinical success assessment

In this study, the following criteria were used to report the outcome of this TAT procedure. Tooth survival was defined as the presence of transplanted tooth still in situ and without associated pain reported during the telephone survey. Teeth associated with symptoms or having been removed were classified as failure at this first stage of the analysis. For teeth undergoing follow-up examination, we considered the procedure clinically successful when the transplant was present with: 1-normal masticatory function; 2-a physiologically sufficient, healthy, and stable periodontium; 3-physiological mobility; 4-normal percussion sound; 5-probing depths < 3 mm; 6- no signs of inflammation; 7-no percussion pain; 8-no discomfort.

Radiological criteria are 1-the presence of a normal alveolar bone process, 2-a normal PDL space with the presence of a lamina dura, 3-no evidence of root resorption or periapical, intra- or periradicular radioluclency, 4-no evidence of ankylosis and 5progressive obliteration of the pulp canal in TAT without root canal treatment.

-Statistical analysis

The statistical analysis included a descriptive analysis of the population, consisting of the mean, the median and standard deviation for all variables. However, there was not enough material for a complete statistical analysis.

## Results

-Patient Selection

The study included 24 patients with 26 transplanted teeth, employing a two-stage surgical technique with double periodontal stimulation and 3D replicas. Four patients (4 donor teeth) considered “lost to follow-up”, leaving 20 patients (55% male, 45% female) with 22 transplanted teeth for evaluation. The median age at surgery was 15 years. Transplants addressed various issues, with the second premolar and maxillary central incisor being common donor and recipient sites, respectively ( Table 1). Fourteen donor teeth were fully erupted, three partially, and five impacted. Nine of the 22 transplants were immature at surgery, while 13 had complete root length and underwent root canal treatment. All recipient sites were prepared with 3D replicas. The average extra-alveolar time (EAT) was under 1 minute, except for two cases with 5 and 3 minutes. Nineteen patients (21 transplants) attended the follow-up examination.

-Survival Analysis

Out of 22 transplants, one failed due to chronic periapical inflammation after 16 months. The overall survival probability was 95.5% (21 of 22 teeth) after a median observation period of 23 months. Mature teeth showed 100% survival after 27 months, while immature teeth exhibited 88.8% survival after 20 months.

-Clinical and Radiological Evaluation

Nineteen patients with 21 transplants and one failed transplant attended the follow-up. Clinical evaluation revealed asymptomatic transplants, with no pathological periodontal pocket depth, recession, bleeding on probing, or abnormal mobility. Dental restorations varied, and all teeth had occlusal contact, with normal masticatory function in all patients. Retroalveolar X-rays showed no measurable bone loss in transplants, with 81% displaying bone level growth. Immature transplants required root canal treatment in two cases, while postoperative root growth occurred in some incomplete root formation stages. Pulp cavity obliteration was observed in immature transplants without endodontic treatment. Favorable PDL healing was recorded in 95% of transplants, and no evidence of root resorption or periapical pathology was found.

-Success Analysis:

The overall success probability, considering clinical and radiographic signs, was 90.5% (19 of 21 transplants) after a median follow-up of 25.5 months. All patients expressed complete satisfaction with the treatment outcome and approach choice.

## Discussion

The survival and success rates reported in the literature differ considerably between authors and for different donor teeth and recipient sites. This inconsistency is likely due to multiple factors that influence treatment, including the surgical protocol. The aim of this study was to evaluate the survival and success rates of TAT by an original surgical protocol predictable in two stages with double periodontal stimulation and the use of 3D replica.

The two-stage technique has shown very good results in decreasing the risk of root resorption, ankylosis and other complications leading to extraction, including mature teeth. This two-step technique, initially described by Nethander *et al*., involves the recipient site being surgically prepared prior to transplantation and allowed to heal for 14 days (15). In contrast, in the one-time technique, the recipient site is prepared and transplantation is performed during the same surgical procedure. Studies have shown that the two-step technique leads to a higher complete healing than the one-time technique, although the difference is not significant (2,5,16)

Furthermore, the contribution of double periodontal ligament stimulation to this two-stage technique has demonstrated the high and rapid regenerative capacity of PDL cells in regenerating periodontal structures. In our protocol, the mechanical stimulation of fibroblasts in the 22 transplanted teeth was achieved by luxation with rotational movements using an elevator, specifically avoiding pendulum movements as they can crush the PDL. This periodontal stimulation also has the advantage of facilitating the extraction of the donor tooth, especially multi-rooted teeth, due to the inflammation of the PDL (17). In vitro and *in vivo* studies also demonstrate that the periodontal ligament fibroblast is the primary cell type involved in socket healing. Mechanical forces and movements will stimulate fibroblast activity and inhibit osteoblasts (18,19). In this protocol, the transplanted tooth is secured in place by sutures to the periodontium and never with a splint connected to other teeth. Thus, under physiological conditions such as chewing or swallowing, the coronal pressure induces limited and episodic movements of the transplanted teeth within the socket.

All 22 recipient sites were prepared during the first surgical stage using a 3D replica of the donor tooth designed from the CBCT, resulting in an average EAT of less than 1 minute. Prolonged EAT has been recognized as a potential iatrogenic lesion to the periodontal ligament (PDL). Although there is no solid evidence published to date regarding the optimal duration of extra-alveolar preservation, a decrease in EAT is recognized as a means of preserving the vitality of PDL cells and improving the prognosis of the tooth transplant. Comparative studies by Shahbazian *et al*. (20) and EzEldeen *et al*. (10) achieved equivalent EAT results, always below 1 minute, with the use of replicas. In our study, 2 transplanted teeth had EAT > 3 minutes. This prolonged EAT could be attributed to the time gap between the CBCT and surgery or the accuracy of the replicas. Verweij *et al*. noted that when using their replicas, the longest EAT and the need for multiple attempts were due to low-quality CBCT images or root development during a long-time interval between CBCT and intervention (21). They recommend performing autotransplantations of immature teeth within 2 months following the CBCT. Studies on the accuracy of dental replicas have identified measurement differences that were deemed clinically accepTable when comparing natural teeth to their radiographic images and printed or machined replicas. Qualitative analysis indicated that the replicas were generally slightly larger in size, which can be beneficial in this clinical context as the transplanted tooth should not be inserted into the recipient site with excessive pressure (22).

This study observed excellent periodontal ligament healing in examined teeth, without root resorption or ankylosis. The majority of transplanted teeth exhibited complete root development, a positive indicator for successful outcomes. Root development stage is a critical prognostic factor for autotransplantation success, with open-apex teeth showing higher success rates. Recent reviews reported over 95% success for open-apex teeth and a 90% survival rate at 5 years for mature transplants. The management of immature transplants differs, with a higher likelihood of pulp regeneration. Root canal treatment is systematically performed for mature teeth, improving their prognosis. Studies show no difference in pulp healing between one-stage and two-stage techniques in our protocol (2).

Pulp canal obliteration and continuous root development were crucial indicators for diagnosing pulp survival. Complete pulp obliteration was observed in all examined immature teeth. Histological examination revealed no signs of inflammation in asymptomatic teeth with pulp obliteration. It’s noteworthy that radiographic obliteration doesn’t guarantee the absence of pulp tissue, potentially indicating ongoing revascularization and dentinogenic cell layer formation within three months (23). Based on these findings, canal treatment is recommended only in the presence of clinical symptoms, negative sensitivity tests, and periapical pathology indicating pulp necrosis. A negative sensitivity test alone is not indicative of pulp necrosis, making pulp obliteration a positive sign of pulp health, eliminating the need for canal treatment (24).

The difference between bone levels, observed before and after surgery, showed the ability of a transplanted tooth with the double stimulation of PDL to regenerate its periodontal support. Another Gault study showed in 47 cases the high potential of stimulated PDL to regenerate alveolar and periodontal bone structures in sites of severe destruction. Indeed, a vertical bone gain (7.73 ± 4.32 mm) was observed, about 2 to 3 times greater than those of other periodontal pocket treatment techniques (6).

In the literature, it has been reported that the survival and success rates of TAT in a natural bone alveolus are higher than those of neoalveoli formed during agenesis (25). However, our two-step technique, which is based on dual periodontal stimulation, yielded excellent results for cases where the creation of a new recipient site in a toothless ridge was necessary. This difference highlights the importance of stimulating periodontal ligament cells at the root level of transplanted teeth.

Despite the interesting methodological aspects of this retrospective study, such as a single center and a standardised treatment procedure, the small sample does not allow any statistical analysis with the reported variables to highlight any correlation effect. The complications for instance, were limited and did not prevent the function but probably the long-term prognosis. We encountered a case of mobility and we lost a case due to acute infection. We did not encounter any case of ankylosis.

Further long-term studies with larger numbers of patients would be needed to confirm the results of the present study. These studies should also detail the indications for transplantation: risk factors, such as smoking, ethnicity, socio-economic factors and education, and type of root canal treatment to account for any factors that could lead to complications. However, this is the first clinical study evaluating a new surgical technique in autotransplanted teeth at any stage of development.

The other investigated aspect was the satisfaction of the patient. The 100% of the cohort referred a total satisfaction and referred that they would have repeated the experience. Surprisingly also the patient in which the transplant failed, reported the same outcome. All patients appreciated the possibility to use an autologous tooth as substitute of another compromised tooth.

## Figures and Tables

**Table 1:** Distribution of transplanted teeth (n = 22) according to their recipient site.

Donor site	Recipient site												
	11	21	33	43	35	16	26	27	36	46	Total
12		1									1
32		1									1
43				1							1
24									1		1
25		1			1						2
15	1	1									2
35		3									3
45	2		1								3
18						1					1
28							1	1		1	3
38										2	2
48										2	2
Total	3	7	1	1	1	1	1	1	1	5	22

## Data Availability

The datasets used and/or analyzed during the current study are available from the corresponding author.

## References

[1] Andreasen JO, Paulsen HU, Yu Z, Ahlquist R, Bayer T, Schwartz O (1990). A long-term study of 370 autotransplanted premolars. Part I. Surgical procedures and standardized techniques for monitoring healing. Eur J Orthod.

[2] Nethander G, Skoglund A, Kahnberg KE (2003). Experimental autogenous tooth transplantation in the dog: a comparison between one- and two-stage surgical techniques. Acta Odontol Scand.

[3] Katayama A, Ota M, Sugito H, Shibukawa Y, Yamada S (2006). Effect of proliferating tissue on transplanted teeth in dogs. Oral Surg Oral Med Oral Pathol Oral Radiol Endod.

[4] Ferreira MM, Botelho MFR, Carvalho L, Silva MR, Oliveiros B, Carrilho EVP (2012). Evaluation of dentin formed in autogenous tooth transplantation in the dog: a comparison between one- and two-stage surgical techniques. Dent Traumatol Off Publ Int Assoc Dent Traumatol.

[5] Nethander G (1994). Periodontal conditions of teeth autogenously transplanted by a two-stage technique. J Periodontal Res.

[6] Gault PC, Warocquier-Clerout R (2002). Tooth auto-transplantation with double periodontal ligament stimulation to replace periodontally compromised teeth. J Periodontol.

[7] Al-Rawi B, Hassan B, Vandenberge B, Jacobs R (2010). Accuracy assessment of three-dimensional surface reconstructions of teeth from cone beam computed tomography scans. J Oral Rehabil.

[8] Tsukiboshi M (2002). Autotransplantation of teeth: requirements for predictable success. Dent Traumatol Off Publ Int Assoc Dent Traumatol.

[9] Chung WC, Tu YK, Lin YH, Lu HK (2014). Outcomes of autotransplanted teeth with complete root formation: a systematic review and meta-analysis. J Clin Periodontol.

[10] EzEldeen M, Wyatt J, Al-Rimawi A, Coucke W, Shaheen E, Lambrichts I (2019). Use of CBCT Guidance for Tooth Autotransplantation in Children. J Dent Res.

[11] Cahuana-Bartra P, Cahuana-Cárdenas A, Brunet-Llobet L, Ayats-Soler M, Miranda-Rius J, Rivera-Baró A (2020). The use of 3D additive manufacturing technology in autogenous dental transplantation. 3D Print Med.

[12] Lee SJ, Kim E (2012). Minimizing the extra-oral time in autogeneous tooth transplantation: use of computer-aided rapid prototyping (CARP) as a duplicate model tooth. Restor Dent Endod.

[13] Lee SJ, Jung IY, Lee CY, Choi SY, Kum KY (2001). Clinical application of computer-aided rapid prototyping for tooth transplantation. Dent Traumatol.

[14] Gault P (2013). Transplantation of impacted canines. Orthod Francaise.

[15] Nethander G, Andersson JE, Hirsch JM (1988). Autogenous free tooth transplantation in man by a 2-stage operation technique. A longitudinal intra-individual radiographic assessment. Int J Oral Maxillofac Surg.

[16] Nethander G (2003). Autogenous free tooth transplantation with a two-stage operation technique. Swed Dent J Suppl.

[17] Nakdilok K, Langsa-Ard S, Krisanaprakornkit S, Suzuki EY, Suzuki B (2020). Enhancement of human periodontal ligament by preapplication of orthodontic loading. Am J Orthod Dentofac Orthop Off Publ Am Assoc Orthod Its Const Soc Am Board Orthod.

[18] King GN, Hughes FJ (1999). Effects of occlusal loading on ankylosis, bone, and cementum formation during bone morphogenetic protein-2-stimulated periodontal regeneration in vivo. J Periodontol.

[19] Matsuda N, Yokoyama K, Takeshita S, Watanabe M (1998). Role of epidermal growth factor and its receptor in mechanical stress-induced differentiation of human periodontal ligament cells in vitro. Arch Oral Biol.

[20] Shahbazian M, Jacobs R, Wyatt J, Denys D, Lambrichts I, Vinckier F (2013). Validation of the cone beam computed tomography-based stereolithographic surgical guide aiding autotransplantation of teeth: clinical case-control study. Oral Surg Oral Med Oral Pathol Oral Radiol.

[21] Verweij JP, van Westerveld KJH, Anssari Moin D, Mensink G, van Merkesteyn JPR (2020). Autotransplantation With a 3-Dimensionally Printed Replica of the Donor Tooth Minimizes Extra-Alveolar Time and Intraoperative Fitting Attempts: A Multicenter Prospective Study of 100 Transplanted Teeth. J Oral Maxillofac Surg Off J Am Assoc Oral Maxillofac Surg.

[22] Lee CKJ, Foong KWC, Sim YF, Chew MT (2022). Evaluation of the accuracy of cone beam computed tomography (CBCT) generated tooth replicas with application in autotransplantation. J Dent.

[23] Singh AK, Khanal N, Acharya N, Hasan MR, Saito T (2022). What Are the Complications, Success and Survival Rates for Autotransplanted Teeth? An Overview of Systematic Reviews and Metanalyses. Healthcare.

[24] Martin K, Nathwani S, Bunyan R (2018). Autotransplantation of teeth: an evidence-based approach. Br Dent J.

[25] Bauss O, Zonios I, Rahman A (2008). Root development of immature third molars transplanted to surgically created sockets. J Oral Maxillofac Surg Off J Am Assoc Oral Maxillofac Surg.

